# Effects of Generic versus Non-Generic Feedback on Motor Learning in Children

**DOI:** 10.1371/journal.pone.0088989

**Published:** 2014-02-11

**Authors:** Suzete Chiviacowsky, Ricardo Drews

**Affiliations:** School of Physical Education, Federal University of Pelotas, Pelotas, RS, Brazil; University of California, Merced, United States of America

## Abstract

Non-generic feedback refers to a specific event and implies that performance is malleable, while generic feedback implies that task performance reflects an inherent ability. The present study examined the influences of generic versus non-generic feedback on motor performance and learning in 10-year-old children. In the first experiment, using soccer ball kicking at a target as a task, providing participants with generic feedback resulted in worse performance than providing non-generic feedback, after both groups received negative feedback. The second experiment measured more permanent effects. Results of a retention test, performed one day after practicing a throwing task, showed that participants who received non-generic feedback during practice outperformed the generic feedback group, after receiving a negative feedback statement. The findings demonstrate the importance of the wording of feedback. Even though different positive feedback statements may not have an immediate influence on performance, they can affect performance, and presumably individuals' motivation, when performance is (purportedly) poor. Feedback implying that performance is malleable, rather than due to an inherent ability, seems to have the potential to inoculate learners against setbacks – a situation frequently encountered in the context of motor performance and learning.

## Introduction

Feedback is information generally provided to learners after each trial or group of trials, referring to their movement's pattern or result on the environment, and is considered one of the most important and studied factors affecting the learning of motor skills [1]. Several studies in different lines of research, such as coaching behavior [2]–[5], self-modeling [6], self-controlled feedback [7]–[11], feedback after successful or “good” trials [12]–[14], or also in the form of positive social comparison [15], [16], have shown that feedback can impact learning via its motivational properties, in addition to its already well-known informational functions.

The motivational properties of feedback were also observed when related to conceptions of ability. Considered as knowledge structures, including beliefs about the inherent ability or the changeability of attributes [17], conceptions of ability has received attention as an important factor affecting performance and learning, mainly in the social-cognitive domain. In general, studies have shown that adults, as well as children, can present or be induced towards different views considering conceptions of abilities. These views can consider abilities as: fixed capacities (entity theorists), in this way defining limits of improvement, or malleable skills (incremental theorists), with improvement, in this way, being strongly dependent of effort and learning [18]–[20]. More specifically, some of these studies have shown that language, in the form of feedback, has the potential to impact children's conceptions of ability, with consequences in their judgments about the stability of a determined personal characteristic over time or contexts, and reflections in its performance and learning. Gelman et al [21] findings showed that children are assumed to be able to judge characteristics of other people as significantly more stable in time when the characteristic was referred to as a noun (e.g., she is an early-waker), as compared with a verbal predicate (e.g., she wakes up early whenever she can). Based on these results, Cimpian et al [22] observed that children are also sensitive to different kinds of praise with respect to their own behavior, with their reactions being influenced by the type of praise provided – generic, inducing a stable trait, and non-generic, inducing a less stable, non-generic trait. In the study by Cimpian et al [22], children who received generic feedback that induced an entity conception of ability (implying that task performance reflects an inherent trait, e.g., you are a good drawer) exhibited more helpless behavior regarding self-evaluation and persistence when criticized than those children who received non-generic feedback that induced an incremental view of ability (implying that performance is malleable or acquirable and referring to a specific event, e.g., you did a good job drawing). The authors concluded that generic feedback can result in children thinking in trait terms, with mistakes being interpreted as showing low ability or a negative trait and can, consequently, decrease their motivation.

Only a few studies had examined the influence of conceptions of ability specifically on motor performance and learning contexts until now, and all have used information in the form of instructions in order to induce different views of ability [23]–[25]. In a study by Jourden et al [24], young adults who performed a rotary pursuit task having been previously instructed that task performance would reflect an acquirable skill, showed higher levels of skill acquisition, perceived self-efficacy, and interest in the activity than those participants who performed the task having previously been instructed that task performance would reflect an inherent aptitude. The study by Wulf et al [25] extended these previous results, showing that the learning of a balance task, measured by a delayed retention test, was enhanced by instructions indicating the learnability of the skill instead of inducing it as reflecting an inherent ability, or with no instructions regarding this aspect (control group). More recently, Drews et al [23] generalized these results to children of different ages, while learning a throwing task.

However, it is still unclear whether feedback information, inducing different conceptions of ability, would be able to affect motor performance and learning. Different from the information used in previous studies in the form of instructions, clearly and objectively inducing the distinct ability conceptions, information provided by generic and non-generic feedback carries a much less direct, or more subtle, connotation of the stability or malleability of an individual's attribute. So, the question still remains as to whether generic and non-generic feedback, inducing different conceptions of ability, has the potential to differently affect motor performance and learning in adults or children.

The purpose of the present study was to examine the effects of generic feedback, inducing an entity view of ability, versus non-generic feedback, inducing an incremental view of ability, on children's motor performance and learning. Two experiments were designed. In the first one, children practiced a soccer kicking task while receiving generic or non-generic feedback statements in the first phase of practice. This was followed by negative feedback statements in the second phase of practice. In order to verify differences in performance between the generic feedback (G-FB) and non-generic feedback (NG-FB) groups, as a function of the type of feedback provided, a retention test was applied 10 min after practice. In the second experiment, we extended the results of the previous one by using a different task – throwing beanbags at a target – and, more importantly, tested possible effects on motor learning. As we wanted to evaluate motor learning in two different situations, following or not following setbacks, all participants wore opaque goggles in all experimental phases in order to avoid intrinsic veridical feedback regarding accuracy scores, and two delayed retention tests were applied, with all participants receiving an equal negative feedback statement after the first retention test. Considering the results of previous studies [22]–[25], we hypothesized that the non-generic feedback groups would demonstrate more effective performance and learning on both tasks, especially when exposed to error or mistakes, than the generic feedback groups.

## Experiment 1

In this experiment, two groups of children received different kinds of feedback, generic or non-generic, while practicing soccer kicks at a target. As in the study by Cimpian et al [22], an important criterion to demonstrate possible different effects was children's reactions to setbacks which, in the present case, were induced by fabricated negative feedback statements, immediately after the first practice phase. If feedback inducing different conceptions of ability has the potential of affect children's behavior, the non-generic feedback group should perform more effectively following the negative feedback statement, and on the immediate retention test, than the generic feedback group.

### Methods

#### Ethics Statement

Written consent was obtained from the parents/guardians, and verbal consent was obtained from the children. The Institutional Review Board of Federal University of Pelotas approved all procedures and the study was conducted according to its regulations.

#### Participants

Forty 10-year-old children (10 females, 30 males; M = 10.0 years, SD  =  0.32) recruited from a city-center private school located in the south of Brazil, without mental or physical disabilities, participated in the study. All of the participants were naive as to the purpose of the experiment. Informed consent was obtained from the school, as well as from the parents/guardians and, in addition, assent was obtained from the participants. The study was approved by the Institutional Review Board of Federal University of Pelotas.

#### Apparatus and Task

**Figure 1:**
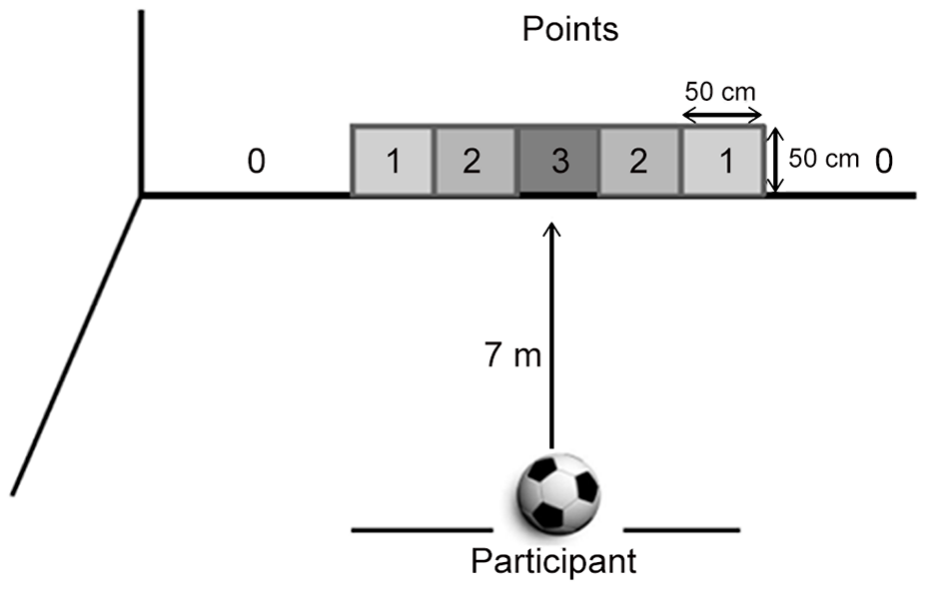
Schematic of the experimental set-up and zone areas used for punctuation.

A regulation-size soccer ball made of leather (circumference: 69 cm; weight: 440 g) was used. The task required participants to perform low kicks at a squared target area consisting of a piece of colored cardboard (measuring 50 cm wide and 50 cm high) attached to a wall and touching the ground, and placed at a distance of seven meters from the participants (Figure 1). The participant's goal was to kick the soccer ball so that it hit the square, which yielded a score of 3 points. Two other zones with the same-sized dimensions were drawn, to the left and right of the target. If the ball hit one of these lateral zones to the left or right side of the target, or missed the target area completely, fewer points were given (2, 1, 0).

#### Procedure

Participants were quasi-randomly assigned to the different groups – generic feedback (G-FB) and non-generic feedback (NG-FB) – considering an equal number of females and males in each group (5 girls and 15 boys). They were informed about the goal of the task and were instructed to kick the ball with their preferred foot.

During the first phase of the experiment, all participants performed 12 trials of kicking the soccer ball at the target. After every third trial, different feedback statements were provided, implying either an inherent ability in the G-FB group (e.g., “You are a great soccer player”; “You have a talent for soccer”) or a malleable skill in the NG-FB group (e.g., “Those kicks were very good”; “The last kicks were great”). Both groups then performed 6 more trials with negative feedback after every third trial (e.g., “Those kicks were not very precise”). This negative feedback was identical for both groups. An immediate retention test, consisting of 6 trials without feedback, was performed 10 minutes later.

#### Data analysis

Accuracy scores were analyzed in 2 (group: G-FB versus NG-FB) x 4 (blocks of 3 trials) analysis of variance (ANOVA) with repeated measures on the last factor for the first experimental phase, and in 2 (group: G-FB versus NG-FB) x 2 (blocks of 3 trials) analysis of variance (ANOVA) with repeated measures on the last factor for the negative feedback phase and the retention test. An alpha level of .05 was used as the threshold for significance.

### Results

#### Phase One

**Figure 2:**
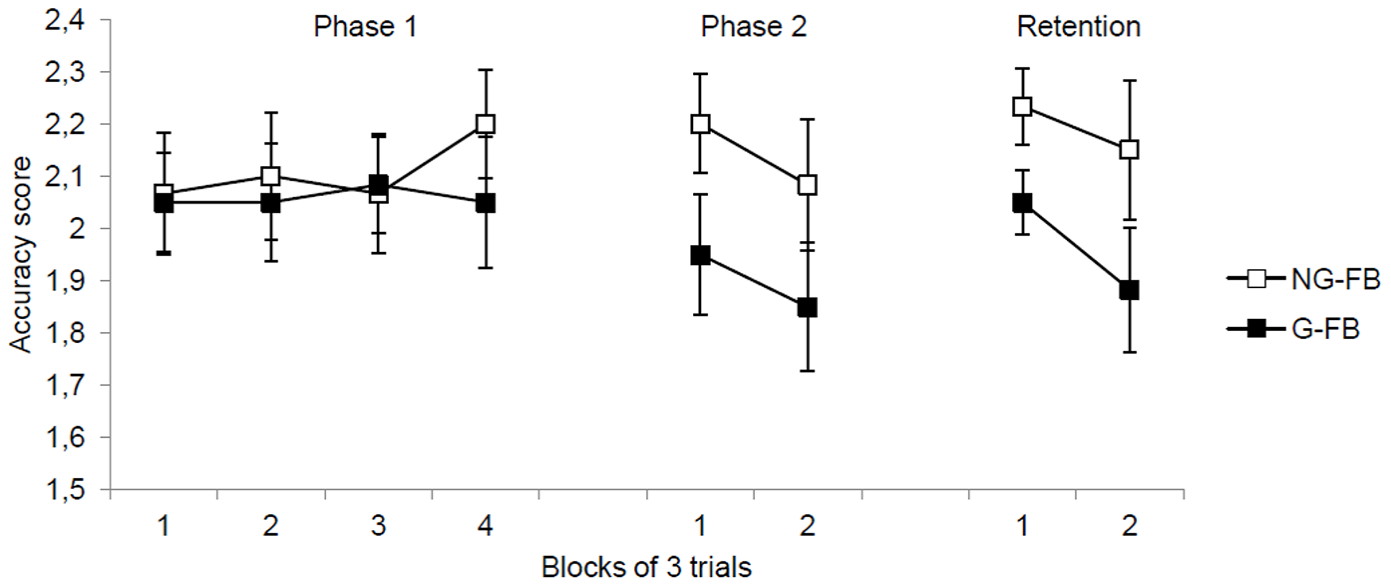
Accuracy scores during phase one, phase two, and retention for the G-FB and NG-FB groups. Error bars indicate standard errors.

Both groups performed with similar accuracy scores across practice blocks (see Figure 2, left side). The main effect of block, *F* (3, 114) <1, and group, *F* (1, 38) <1, were not significant, as well as the interaction of group and block, *F* (3, 114) <1.

#### Phase Two

On the second phase, the NG-FB group presented higher accuracy scores than the G-FB group (see Figure 2, middle). The difference between groups was significant, with *F* (1, 38) = 4.17, *p*<.05, η^2^ = .10. The main effect of block, *F* (1, 38) <1, and the interaction of the block and group, *F* (1, 38) <1, were not significant.

#### Retention test

On the no-feedback retention test, 10-min. later, the NG-FB group also had higher accuracy scores than the G-FB group (see Figure 2, right). The difference between groups was significant, with *F* (1, 38) = 4.39, *p*<.05, η^2^ = .10. The main effect of block, *F* (1, 38) = 1.74, *p*>.05, and the interaction of the block and group, *F* (1, 38) <1, were also not significant.

### Discussion

The findings show that children's motor performance can be affected by the kind of augmented feedback provided during practice: generic, implying stability/inherent trait or non-generic, implying malleability/acquirable skill perception. While there were no differences in shot accuracy between groups in the first phase, the NG-FB group outperformed the G-FB group in the second phase and on the immediate retention test. Thus, generic feedback can degrade not only intrinsic motivation in children [22] but also motor performance, when compared with non-generic feedback.

## Experiment 2

The second experiment was designed to investigate more permanent effects of providing non-generic versus generic feedback during practice in children, through the use of an extended practice phase and delayed retention tests. In addition, we wanted to test the generalization of the effect observed in experiment one on a different task – throwing beanbags at a target. Lastly, since the effect of the negative feedback statements could have been diluted somewhat by the knowledge of the participant's actual performance through intrinsic visual feedback in the previous experiment, we prevented participants from viewing the target during practice and both retention tests. During practice, half of the participants received generic feedback, implying an inherent ability, while the other half received non-generic feedback, implying the task as an acquirable skill. One day later, motor learning was verified, as a function of the type of feedback provided during practice, on two retention tests, with the second test measuring participants reactions to a setback induced by an identical fabricated negative feedback, provided immediately after the completion of the first test. Taking into account the results of the first experiment of the present study as well as previous studies on the subject [22]–[25], we expected that the non-generic feedback group would outperform the generic feedback group on both retention tests.

### Methods

#### Ethics Statement

Written consent was obtained from the parents/guardians, and verbal consent was obtained from the children. All procedures were approved and conducted according to the regulations of the Institutional Review Board of Federal University of Pelotas.

#### Participants

Forty 10-year-old children (20 females, 20 males; M = 10.5 years, SD = 0.51), without mental or physical disabilities, participated in the study. The participants were naive as to the purpose of the experiment and none of them had participated in the previous experiment. As in experiment 1, they were recruited from a city-center private school located in the south of Brazil.

#### Apparatus and Task

The task – the same as used in a previous study [26] – involved participants throwing beanbags (100 g) at a circular target placed on the floor, with their non-dominant arm, and while wearing opaque goggles. The center of the target was placed at a distance of 3 m from the participant, and the accuracy scores were based on where the beanbag first contacted the floor. When the beanbag landed on the bull's eye, 100 points were awarded. If it landed outside the circles, 90, 80, 70 … 0 points, respectively, were recorded. The higher score was awarded if a beanbag landed on a line. The target had a radius of 10 cm.

#### Procedure

Participants were quasi-randomly assigned to the generic feedback group (G-FB) and to the non-generic feedback group (NG-FB), with an equal number of males and females in each group (10 boys and 10 girls). In order to prevent them from viewing the target during practice and both retention tests, the participants wore opaque swimming goggles while throwing. However, they were allowed to look at the target before all experimental phases. The children were informed about the goal of the task and were instructed to throw the beanbags overhand with the non-dominant hand, while keeping their feet on the floor and behind a line.

**Figure 3:**
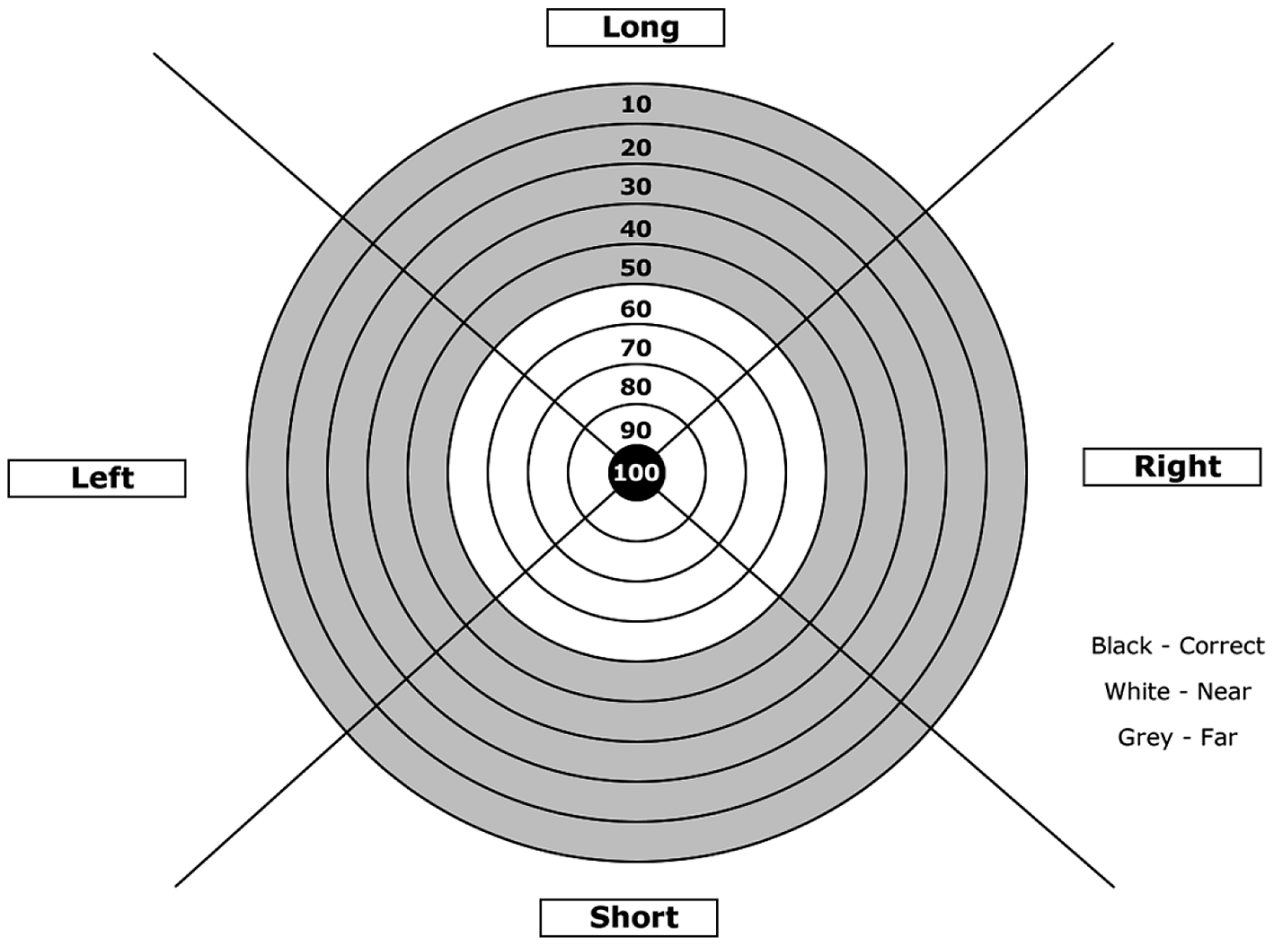
Target and zone areas used for providing feedback.

Participants performed 40 trials in the practice phase. After each block of 10 trials, different feedback statements were provided, implying an inherent ability (e.g., “You have talent for throwing”) in the GF group, or a malleable skill (e.g., “These last throws were very good”) in the NGF group. In addition, participants also received veridical feedback regarding throwing accuracy after each trial. The target area was divided into four quadrants in order to provide directional information (Figure 3). These areas were designated as “long”, “short”, “left”, or “right”. Feedback included information about the direction and the distance from the center of the target (e.g., “a little bit to the right” or “much too long”) depending on whether the beanbag landed in the inner (60–100) or outer circles (0–50), respectively.

One day later, both groups performed two retention tests, consisting of 10 trials each, with vision occluded and without veridical feedback related to accuracy scores. After the first retention test, all participants were provided with one (identical) negative statement: “On these last throws, you did not do very well”. This was followed by the second retention test.

#### Data analysis

Accuracy scores in the practice phase were analyzed in 2 (group: G-FB versus NG-FB) x 4 (blocks of 10 trials) analysis of variance (ANOVA), with repeated measures on the last factor. For both retention tests, accuracy scores were analyzed in separate one-way ANOVAs. Alpha level for significance was set at .05 for all analyses.

### Results

#### Practice

**Figure 4:**
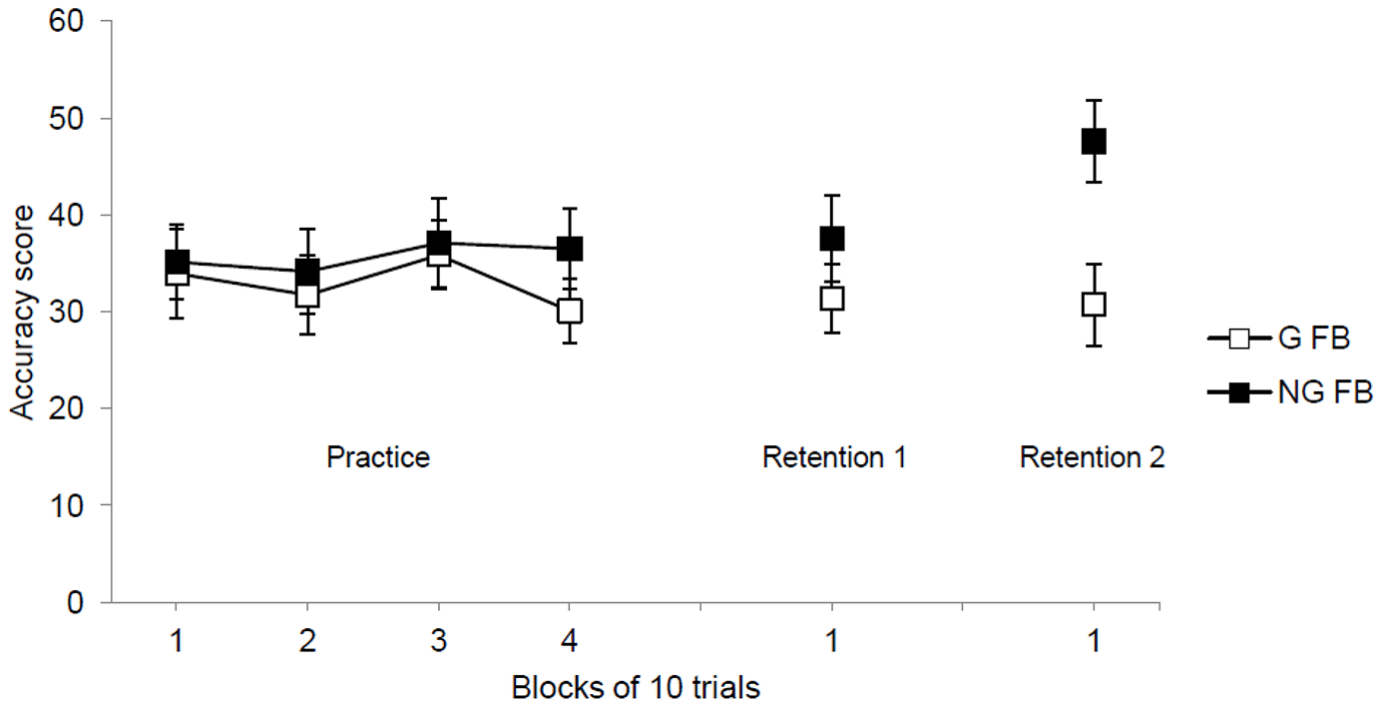
Accuracy scores during practice and retention tests for the G-FB and NG-FB groups. Error bars indicate standard errors.

Both the G-FB and NG-FB groups performed with similar accuracy scores across practice blocks (see Figure 4, left side). The main effect of block, *F* (3, 114) <1, group, *F* (1, 38) <1, as well as the interaction of group and block, *F* (3, 114) <1, were not significant.

#### Retention One

On the first retention test, performed 1 day after the practice phase, the NG-FB group presented slightly higher accuracy scores than the G-FB group (see Figure 4, middle). However, the difference between groups was not significant, with *F* (1, 38) = 1.20, *p*>.05.

#### Retention Two

On the second retention test, after the negative feedback statement, the NG-FB group had higher accuracy scores than the G-FB group (see Figure 4, right). The main effect of group was significant, *F* (1, 38) = 8.12, *p*<.01, η^2^ = .17.

### Discussion

The findings of the second experiment demonstrate that feedback, inducing different conceptions of ability during practice, can present more permanent effects that affect children's motor learning. Results of a retention test, performed one day after practicing the throwing task, showed that participants which received non-generic feedback during practice outperformed the generic feedback group after receiving a negative feedback statement. While participants of the G-FB group maintained their accuracy relative to the first retention test, participants of the NG-FB group tended to improve it. *Thus, different* positive feedback statements may not have an immediate influence on performance, but they can affect performance, and presumably individuals' motivation, when performance is poor. In the same direction as discussed by Dweck [19] and Dweck et al [20], non-generic feedback has the potential to make children put in more effort after errors or mistakes, not seeing the negative feedback information as a threat to the self, and in this case decreasing performance, when compared with generic feedback, and these effects seems to be relatively permanent.

## General Discussion

Recent studies have shown that feedback can affect motor learning, not only by its already well-established informational function, but also by a motivational one, both in adults [7]–[9], [12], [16], [27], [28] and children [14], [15], [26]. The general objective of this research was to examine the effects of generic versus non-generic feedback on motor performance and learning of motor skills in children. The first study was more exploratory, and aimed at testing the effects of these different types of feedback on children's motor performance. The objective of the second study was to verify more permanent effects of generic versus non-generic feedback on motor learning in children, as well as to generalize the results for a different task. For both studies, we hypothesized that participants receiving non-generic feedback would outperform participants receiving generic feedback, especially after setbacks.

Our hypothesis was confirmed. While the provision of positive feedback, in general, has been viewed as a good strategy to promote motor learning, the present findings show that some kinds of positive feedback may not work as desired, especially when individuals need to cope with setbacks. Participants of the present study who received generic feedback, inducing the task as reflecting inherent ability conceptions, underperformed participants receiving learnable ability conceptions, with effects being demonstrated not only on motor performance, but also on motor learning. So, the present results extend previous motivational findings with children [22] by showing that the detrimental effects of generic feedback also play out in motor performance and learning contexts. The generic and non-generic feedback statements provided during practice for the different groups seem to have the potential to induce different conceptions of ability in children, with consequences on their learning, similarly to what happens when the different conceptions are induced more directly through instructions, in children [23] and adults [25].

However, in contrast with previous studies which induced conceptions of ability by instructions [23], [25], the effects of conceptions of ability induced by feedback on motor learning did not appear on the first regular delayed retention test, but only on the second retention test, after the negative feedback statement. The more subtle connotation presented by generic feedback, emphasizing abilities as fixed capacities, or in non-generic feedback, inducing the malleability of abilities, apparently impact motor learning in a different way, when compared with the more direct information provided in the form of instructions. Anyway, the present results are in agreement with the view that entity theorists strive to “prove” their abilities by outperforming others, while incremental theorists seem to be focused on an effort to “improve” on the task, tending to be more intrinsically motivated (for a review see Burnette et al [29]). In this view, there are indications showing that while entity theorists tend to avoid challenging situations, since they might demonstrate low ability, and demonstrate less persistence when confronted with error feedback, incremental theorists tend to react to difficult situations or mistakes by increasing their effort, since they can represent a natural part of the learning process [30]–[32].

The mechanisms by which conceptions of ability affect learning have been proposed in the academic context, mainly taking into account how perceived competence is developed. According to Nicholls [32], Ames et al [33] and Dweck [34], individuals can construe competency in two different ways. If competence is construed in a learning involvement context, considering tasks as learnable skills, levels of competence are judged in relation to one's own perceived mastery, with more learning reflecting more competence. On the other hand, if competence is formed in a performance involvement context, where the task reflects an inherent trait, then competence is normally judged with reference to external values or norms. These two different constructions of competence can result in different behavioral reactions, mainly when individuals are presented with negative situations or setbacks. In fact, previous studies have shown that individuals displaying an acquirable skill view continue to seek challenges and present high performance and persistence on the task, while individuals displaying an inherent trait view present a helpless behavior – including an avoidance of challenges and a low persistence on the task, since errors can carry evaluative threats – undermining aspects related to self-assessment and affect [20], [32], [34], [35].

In the motor behavior domain, more specific mechanisms are being proposed as responsible for the observed performance and learning effects of different conceptions of ability. Previous studies already showed that a learnable/malleable view, as opposed to an inherent/fixed view, can be associated with increased self-efficacy [24], reduced nervousness and thoughts about one's own ability, less attention being directed to body movements and greater automaticity in motor control [25]. According to Wulf et al [25], performance conditions that produce low motivational states have the potential to provoke implicit access to the self, which, according to Carver et al [36] usually happens through self-regulatory processes in order to control individual's thoughts and emotions. With this implicit access to the self, the attentional capacity can be exceeded, producing “micro-choking” episodes, consequently undermining motor performance and learning [25]. Thus, the non-generic feedback information provided in the present study during practice may have reduced participants' self-focus especially after receiving negative feedback, thus differentiating performance and learning between groups.

In conclusion, the detrimental effects of negative feedback observed on the performance of participants of the G-FB groups of the present studies can directly demonstrate that the provision of feedback related to a general trait, during practice, is not beneficial for children, and must be avoided during the motor learning process. Generic positive feedback, inducing stable traits, can degrade future motor performance and learning in children when facing setbacks, probably because mistakes can reflect a perceived low ability or competence. On the other hand, non-generic feedback related to a specific process or strategy, inducing malleable or acquirable skill perceptions, can promote a more positive reaction to negative feedback, as reflected here by a more stable performance after errors, in both experiments.

The present results add to the growing evidence of the impact of motivational factors on motor learning, and demonstrate the importance of the wording of feedback. Non-generic feedback implying that performance is malleable, rather than due to an inherent ability, seems to have the potential to inoculate learners against setbacks – which are frequently encountered in the context of motor performance and learning. Future studies could try to examine the motivational role of generic and non-generic feedback more directly (e.g., through the use of questionnaires after the practice phase) in order to gain a better understanding of the mechanisms involved in these different types of feedback during the motor learning process.

## References

[1] Schmidt RA, Lee TD (2011) Motor control and learning: A behavioral emphasis. Champaign, IL: Human Kinetics.

[2] Allen JB, Howe BL (1998) Player ability, coach feedback, and female adolescent athletes' perceived competence and satisfaction. J Sport Exerc Psychol 20: 280–299.

[3] Amorose AJ, Horn TS (2000) Intrinsic motivation: Relationships with collegiate athletes' gender, scholarship status, and perceptions of their coaches' behavior. J Sport Exerc Psychol 22: 63–84.

[4] Koka A, Hein V (2003) The impact of sports participation after school on intrinsic motivation and perceived learning environment in secondary school physical education. Kinesiology 35: 5–13.

[5] Mouratidis M, Vansteenkiste M, Lens W, Sideridis G (2008) The motivating role of positive feedback in sport and physical education: Evidence for a motivational model. J Sport Exerc Psychol 30: 240–268. 18490793 10.1123/jsep.30.2.240

[6] Clark SE, Ste-Marie D (2007) Self as a model: psychological and physical performance benefits. J Sports Sci 25: 577–586. 17365543 10.1080/02640410600947090

[7] Chiviacowsky S, Wulf G (2002) Self-controlled feedback: Does it enhance learning because performers get feedback when they need it? Res Q Exerc Sport 73: 408–415. 12495242 10.1080/02701367.2002.10609040

[8] Chiviacowsky S, Wulf G (2005) Self-controlled feedback is effective if it is based on the learner's performance. Res Q Exerc Sport 76: 42–48. 15810769 10.1080/02701367.2005.10599260

[9] Chiviacowsky S, Wulf G, Lewthwaite R (2012) Self-controlled learning: The importance of protecting perceptions of competence. Front Psychol 3: Article 458. doi:10.3389/fpsyg.2012.00458. PMC3487418 23130006

[10] Fairbrother JT, Laughlin DD, Nguyen TV (2012) Self-controlled feedback facilitates motor learning in both high and low activity individuals. Front Psychol 3: Article 323. doi:10.3389/fpsyg.2012.00323. PMC3431613 22969745

[11] Patterson JT, Carter M (2010) Learner regulated knowledge of results during the acquisition of multiple timing goals. Hum Mov Sci 29: 214–27 10.1016/j.humov.2009.12.003 20338655

[12] Chiviacowsky S, Wulf G (2007) Feedback after good trials enhances learning. Res Q Exerc Sport 78: 40–47. 17479573 10.1080/02701367.2007.10599402

[13] Chiviacowsky S, Wulf G, Wally R, Borges T (2009) Knowledge of results after good trials enhances learning in the elderly. Res Q Exerc Sport 80: 663–668. 19791654 10.1080/02701367.2009.10599606

[14] Saemi E, Wulf G, Varzaneh AG, Zarghami M (2011) Feedback after good versus poor trials enhances learning in children. Rev Bras Educ Fís Esporte 25: 671–679.

[15] Ávila LTG, Chiviacowsky S, Wulf G, Lewthwaite R (2012) Positive social-comparative feedback enhances motor learning in children. Psychol Sport Exerc 13: 849–853 10.1016/j.psychsport.2012.07.001

[16] Lewthwaite R, Wulf G (2010) Social-comparative feedback affects motor skill learning. Q J Exp Psychol 63: 738–749 10.1080/17470210903111839 19691002

[17] Ross M (1989) Relation of implicit theories to the construction of personal histories. Psychol Rev 96: 341–357.

[18] Dweck CS (1999) Self-theories: Their role in motivation, personality, and development. Philadelphia: Psychology Press.

[19] Dweck CS (2002) The development of ability conceptions. In: Wigfield A, Eccles JS, editors, Development of achievement motivation. pp. 57–88. New York: Academic.

[20] Dweck CS, Leggett EL (1988) A social-cognitive approach to motivation and personality. Psychol Rev 95: 256–73.

[21] Gelman SA, Heyman GD (1999) Carrot-eaters and creature-believers: The effects of lexicalization on children's inferences about social categories. Psychol Sci 10: 489–493.

[22] Cimpian A, Arce HM, Markman EM, Dweck CS (2007) Subtle linguistic cues affect children's motivation. Psychol Sci 18: 314–316. 17470255 10.1111/j.1467-9280.2007.01896.x

[23] Drews R, Chiviacowsky S, Wulf G (2013) Children's motor skill learning is influenced by their conceptions of ability. JMLD 1: 38–44.

[24] Jourden FJ, Bandura A, Banfield JT (1991) The impact of conceptions of ability on self-regulatory factors and motor skill acquisition. J Sport Exerc Psychol 8: 213–226.

[25] Wulf G, Lewthwaite R (2009) Conceptions of ability affect motor learning. J Mot Behav 41: 461–467 10.3200/35-08-083 19491058

[26] Chiviacowsky S, Wulf G, Medeiros F, Kaefer A, Tani G (2008) Learning benefits of self-controlled knowledge of results in 10-year old children. Res Q Exerc Sport 79: 405–410. 18816953 10.1080/02701367.2008.10599505

[27] Badami R, VaezMousavi M, Wulf G, Namazizadeh M (2011) Feedback after good trials enhances intrinsic motivation. Res Q Exerc Sport 82: 360–364. 21699117 10.1080/02701367.2011.10599765

[28] Saemi E, Porter JM, Varzaneh AG, Zarghami M, Maleki F (2012) Knowledge of results after relatively good trials enhances self-efficacy and motor learning. Psychol Sport Exerc 13: 378–382.

[29] Burnette JL, O'Boyle EH, VanEpps EM, Pollack JM, Finkel EJ (2013) Mind-sets matter: A meta-analytic review of implicit theories and self-regulation. Psychol Bull 139: 655–701 10.1037/a0029531 22866678

[30] Hong Y, Chiu C, Dweck CS, Lin D, Wan W (1999) Implicit theories, attributions, and coping: A meaning system approach. J Pers Soc Psychol 77: 588–599.

[31] Martocchio J (1994) Effects of conception of ability on anxiety, self-efficacy, and learning in training. J Appl Psychol 79: 819–825. 7852207 10.1037/0021-9010.79.6.819

[32] Nicholls JG (1984) Achievement motivation: Conceptions of ability, subjective experience, task choice and performance. Psychol Rev 91: 328–346.

[33] Ames C, Archer J (1988) Achievement goals in the classroom: Students' learning strategies and motivation processes. J Educ Psychol 80: 260–267.

[34] Dweck CS (1986) Motivational processes affecting learning. Am Psychol 41: 1040–1048.

[35] Kamins ML, Dweck CS (1999) Person versus process praise and criticism: Implications for contingent self-worth and coping. Dev Psychol 35: 835–847. 10380873 10.1037//0012-1649.35.3.835

[36] Carver CS, Scheier MF (1978) Self-focusing effects of dispositional self-consciousness, mirror presence, and audience presence. J Pers Soc Psychol 36: 324–332.

